# Song Circuit in Bird Brain Contains Map of Space and Time

**DOI:** 10.1371/journal.pbio.1002159

**Published:** 2015-06-03

**Authors:** Janelle Weaver

**Affiliations:** Freelance Science Writer, Carbondale, Colorado, United States of America

**Fig 1:**
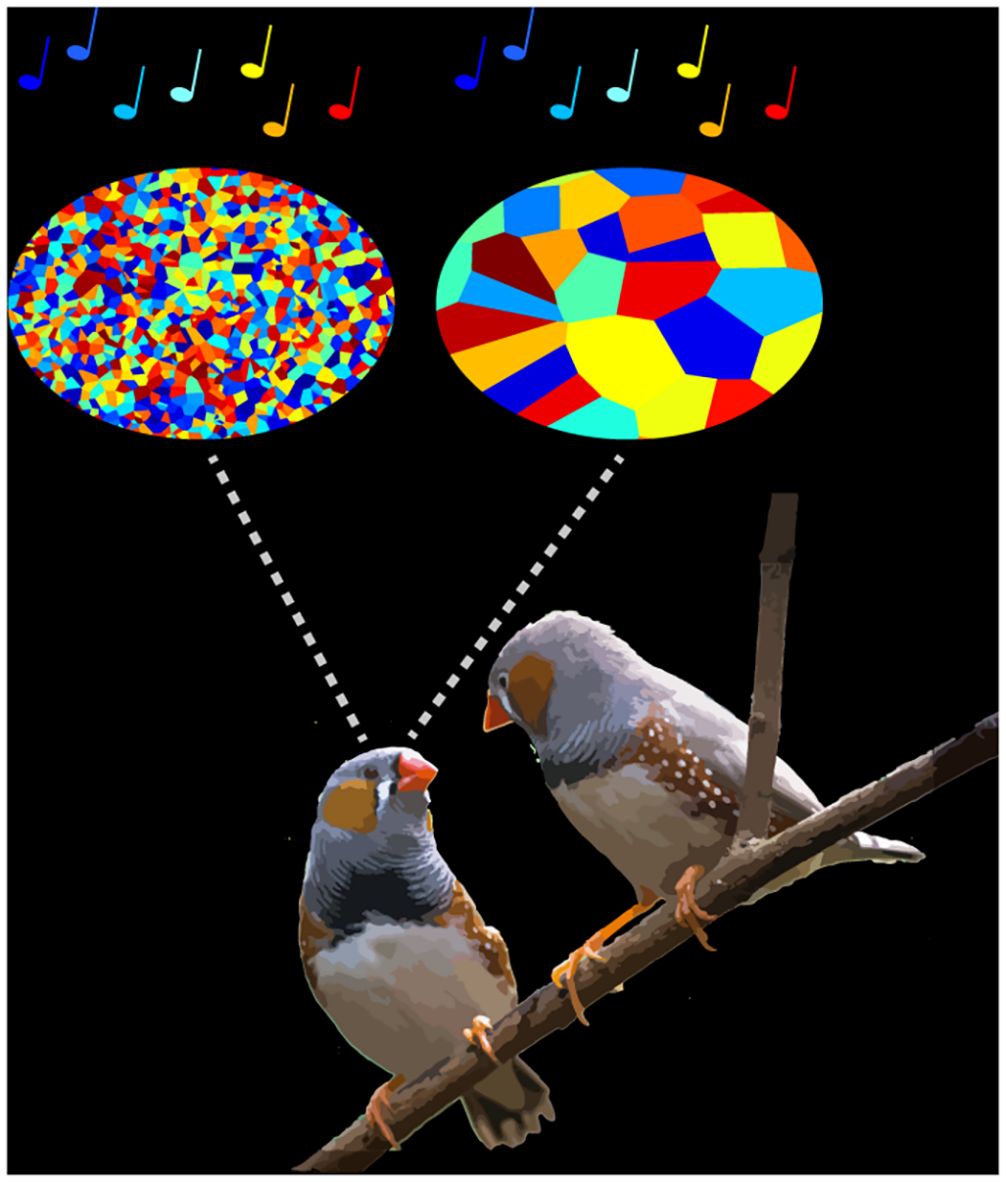
Is time represented with spatially disorganized (left) or locally organized (right) structure? Optical and electrical measurements in singing birds reveal the latter. *Image credit*: *Jeffrey Markowitz; birds adapted from NeilsPhotography*, *Flickr*.

Many brain regions involved in perception and movement are characterized by orderly maps, with neighboring neurons responding to similar features of sensory input or motor output. For example, brain circuits involved in motor control contain maps for distinct muscle groups and preferred direction of movement. However, it has not been clear whether higher-order features, such as time in a movement sequence or serial order, are represented topographically (see Fig 1). Even though temporal sequence generation is integral to many forms of learned behavior, the underlying mechanisms are not well understood.

One attractive brain region for addressing the question of how time is mapped in space is the premotor nucleus HVC in songbirds. This brain region plays a crucial role in the learning and production of bird song and is responsible for generating extremely precise, learned temporal sequences. HVC neural activity drives downstream areas controlling the vocal organ and also projects to the basal ganglia, which is thought to guide exploratory trial-and-error learning. However, past technical limitations have prevented scientists from exploring the spatiotemporal organization of HVC activity in singing birds.

In a study published this week in *PLOS Biology*, Timothy Gardner of Boston University and his collaborators provide the first experimental evidence of how HVC ensemble activity is organized in space and time in singing zebra finches. To accomplish this feat, they developed new methods for multi-channel electrophysiology and calcium imaging in singing birds. They found that the activity patterns of HVC neurons are spatially clustered, and that two HVC cell types fire in alternating phases of a 30 Hz rhythm. According to the authors, this spatial and temporal organization may be an evolutionarily conserved feature of motor circuits across species.

Using calcium imaging in freely behaving, singing zebra finches, the researchers examined the spatial organization of the neural activity of projection neurons, which send signals from the HVC to the vocal organ and basal ganglia. They found that neighboring neurons showed similar patterns of neural activity, but cells that did not fire at the same time were not located close to one another. The spatial correlations in HVC calcium activity were characterized by a patchwork of domains organized in stereotyped sequences, with an average distance of 143 μm separating coactive cells.

The researchers next addressed whether inhibitory interneurons in HVC show a similar spatial and temporal organization as projection neurons. Because the timescale of the calcium indicator they used could not resolve the fast firing patterns of the interneurons, they combined multi-channel electrophysiology with local field potentials (LFP) recordings, which can reflect synchronous ensemble neural activity over roughly 100 μm. They found that HVC neurons fired in synchrony about 30 times per second, or 30 Hz. Moreover, LFPs recorded from nearby electrodes were more similar than those recorded from electrodes separated by about 200 μm. Averaging over all recording sites in all birds, they found that synchronous neural activity at 30 Hz occurred across a distance of 108–125 μm. Taken together with the calcium imaging results, these findings indicate that HVC activity during singing is correlated over a distance of roughly 100 μm.

When comparing the timing of activity in the two cell types, the researchers found that projection neurons fire during peaks in the 30 Hz LFP, whereas interneurons fire in the troughs. These observations indicate that the two cell types fire in opposition to generate a local 30 Hz rhythm in the LFP, and that synchronous pauses in inhibition could provide a window of opportunity for projection neurons to fire. Given that little was previously known about the interaction between inhibitory neurons and projections neurons in singing birds, the findings shed new light on the fundamental rules that relate the activity of these two classes of cells.

More broadly, a comparison with past studies reveals commonalities across species and behavioral contexts. For example, the 100 μm length scale observed during singing has also been observed in the motor cortex of mammals in the context of running, grooming, and simple forelimb movements. In addition, the 30 Hz rhythm has parallels in primate motor control as well as speech perception and production in humans. According to the authors, the recurrence of these length scales and timescales raises the possibility that spatial clustering and 30 Hz rhythmic alternation of excitatory and inhibitory neural activity may be a fundamental feature of motor circuits and could be critical for the sequential organization of behavior.
